# Screening of Antioxidant Maillard Reaction Products Using HPLC-HRMS and Study of Reaction Conditions for Their Production as Food Preservatives

**DOI:** 10.3390/molecules29204820

**Published:** 2024-10-11

**Authors:** Sara Bolchini, Roberto Larcher, Ksenia Morozova, Matteo Scampicchio, Tiziana Nardin

**Affiliations:** 1 Faculty of Agricultural, Environmental and Food Science, Free University of Bolzano, 39100 Bolzano, Italy; sbolchini@unibz.it (S.B.); ksenia.morozova@unibz.it (K.M.); matteo.scampicchio@unibz.it (M.S.); 2 Centro di Trasferimento Tecnologico, Fondazione Edmund Mach, 38010 San Michele all’Adige, Italy; tiziana.nardin@fmach.it

**Keywords:** Maillard reaction, antioxidants, food preservatives, high-resolution mass spectrometry

## Abstract

The Maillard reaction (MR) involves interactions between reducing sugars and amino acids or proteins during heating, producing Maillard reaction products (MRPs) that influence food flavour, aroma, and colour. Some MRPs exhibit antioxidant properties, prompting interest in their potential as natural food preservatives. This study aimed to develop a method for detecting and identifying antioxidant MRPs using high-pressure liquid chromatography (HPLC) coupled with high-resolution mass spectrometry (HRMS). By improving chromatographic conditions, the separation of antioxidant MRPs was optimised using known antioxidant MRPs as reference signals. This work also examined the effects of pH, reaction time, and different sugar–amino acid combinations on the production and composition of antioxidant MRPs. Results indicated that neutral to basic pH facilitated faster reactions, with pH 7 selected as optimal. A library of 50 *m/z* signals for potential antioxidant MRPs was created, and the best combinations of amino acids and sugars for their production were identified. These findings pave the way for more precise analyses of antioxidant MRPs, with future research focusing on isolating and characterising specific MRPs to understand their structures and mechanisms, ultimately contributing to the development of functional foods with natural antioxidant properties.

## 1. Introduction

The Maillard reaction (MR) is a complex series of chemical reactions that occur between reducing sugars and amino acids or proteins during heating. This reaction leads to the formation of Maillard reaction products (MRPs) that are responsible for flavours, aromas, and colour formation in food [1,2,3]. Furthermore, some MRPs have been reported to possess antioxidant activity, offering potential health and food maintenance benefits [4,5,6]. Indeed, MR can be considered a potential way to obtain natural antioxidant compounds from widely common molecules, especially reducing sugars and amino acids, present in food and food waste. In the food chain, antioxidant compounds represent an important class of food preservatives because they prevent oxidative deterioration of food, thereby extending its shelf life and maintaining quality.

Moreover, natural food preservatives have gained attention from the scientific community due to increasing concerns about the health impacts of synthetic additives, the rise of the clean label trend, and the growing demand for sustainable food practices. As consumers seek safer and more wholesome options, there is a need for alternative preservation methods derived from natural sources [7]. 

Although the antioxidant activity developed with MR in both model systems and real food has been widely studied using mainly antioxidant assays like the spectrophotometric assay based on 2,2-diphenyl-1-picrylhydrazyl (DPPH) change of colour, ferric reducing power assay (FRAP), or oxygen radical absorbance capacity assay (ORAC) [3,5,8], limited information is available regarding their structure and composition. Moreover, although the influence of important variables like pH, type of reducing sugar, amino compounds, or time of reaction on MR has been extensively studied in previous literature [9], their influence on the production and composition of antioxidant MRPs is not well understood.

Accordingly, the general aim of this work was to define the optimal MR conditions to obtain, on an industrial scale, antioxidant MRPs from solutions of amino acids and sugars, with the objective of producing natural food antioxidants. Initially, an analytical method was developed to enable the detection and, when possible, identification of potential antioxidant MRPs. This was feasible using High-Pressure Liquid Chromatography (HPLC) coupled with High-Resolution Mass Spectrometry (HRMS). After proper optimisation of the chromatographic method, it allowed the separation and detection of a large selection of molecules produced during MR. To develop and optimise the analytical method, HRMS signals corresponding to 10 known antioxidant MRPs (KAMs) were used as references [10,11].

Additionally, the influence of pH, time of reaction, and different sugars and amino acids on the production, composition, and structure of KAMs was studied using the previously set signals as an index to evaluate which conditions produced the maximum yield of KAMs. 

Since MR is very complex and KAMs may not represent all the antioxidants produced during the reaction, it became essential to increase the number of signals studied to evaluate the influence of variables (pH, time, and reagent composition) on the production of additional potential antioxidant MRPs (PAMs). Therefore, through untargeted HPLC-HRMS analyses of a mix of sugars and amino acids at pH 7, a pool of ions corresponding to PAMs was selected and monitored during the reaction with a radical initiator, which caused a decrease in the corresponding signals. Once the signals corresponding to PAMs were selected, their production was monitored in MR samples with different pH, time of reaction, and combinations of common sugars and amino acids in food, allowing for a more comprehensive evaluation of each variable’s influence on the production of PAMs.

## 2. Results and Discussion

### 2.1. Optimisation of Chromatographic Method

The optimisation of the chromatographic method was done using as a reference the signals corresponding to KAMs reported in Table 1. Some compounds were tentatively identified based on *m/z* values and fragmentation profiles (MS^2^) compared with the literature, while for others (indicated in Table 1 with *), the identification was confirmed with standard reagents. For 1-methyl-2-pyrrole-carboxaldehyde and 2-acetyl-1-methylpyrrole, three different peaks were detected. In both cases, all had the same *m/z* and fragmentation patterns, suggesting the isomeric nature of the molecules responsible for the peaks. Since no standard compounds were available, all three peaks were considered in the following integration of areas. This choice was supported by the fact that in the presence of a radical initiator, all three peaks decreased, indicating that the molecules represented by the peaks are potential antioxidants and corroborating the hypothesis that these molecules are isomers.

**Figure 1:**
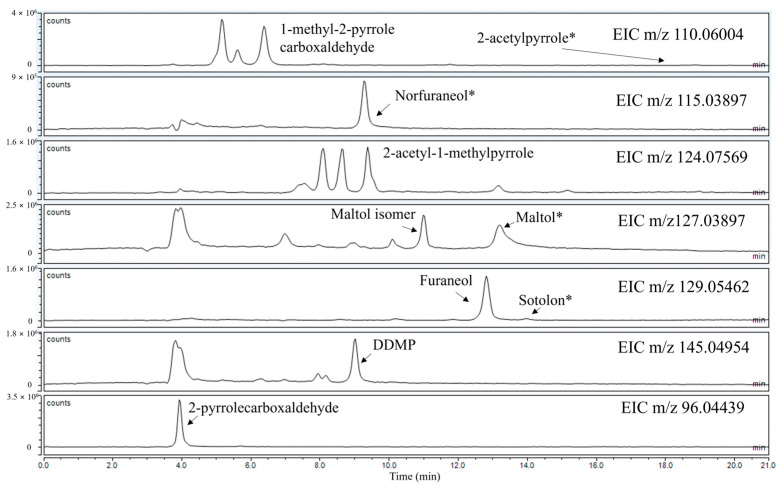
EICs corresponding to the *m/z* of the KAMs considered. (*) indicates the confirmed identifications with standard compounds.

In Figure 1**,** the extracted ion chromatograms (EIC) referring to the *m/z* of the best separation obtained for the KAMs are reported. The same figures obtained with different columns are available in the supporting information (Figure S1).

The final conditions selected after the optimisation of the method and applied in all the subsequent experiments are reported here: the Dionex IonPac NS2 column (4 × 150 mm, 5 µm particle size Thermo Fisher Scientific, USA) was used for the analysis. The column temperature was kept constant at 30 °C. The mobile phases were Milli-Q water with 0.5% formic acid (*v/v*) (A) and acetonitrile with 0.5% formic acid (*v/v*) (B). Before injection, the samples were diluted 1:10 with Milli-Q water, and the injection volume was set to 2 µL. The analysis was conducted at a constant flow rate of 0.35 mL·min^−1^ using the following gradient for optimal chromatographic separation: from 0 to 1 min, 5% of eluent B; from 1 to 10 min, 15% eluent B; from 10 to 15 min, 35% eluent B; from 15 to 21 min, 5% eluent B. 

### 2.2. Influence of Initial pH on Antioxidant Production in MR

**Figure 2:**
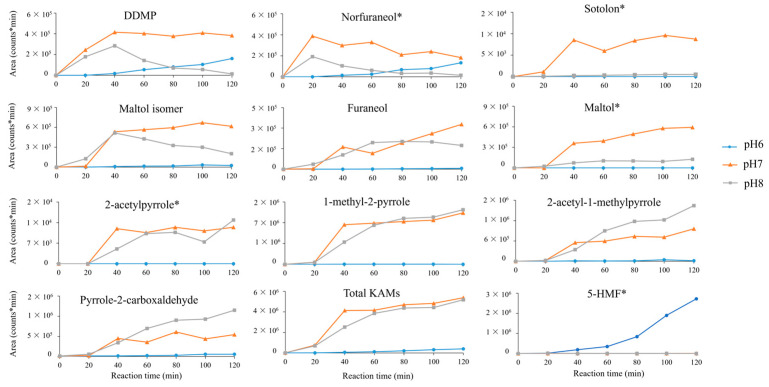
Kinetics of production of 10 KAMs at pH 6, 7, and 8. The sum of the signals of the known antioxidant MRPs (KAMs) and the kinetics of production of 5-hydroxymethylfurfural (HMF) are also reported. Measurements were performed in duplicate with a relative standard deviation (RSD) lower than 10%. (*) indicates the confirmed identifications with standard compounds.

The influence of pH at different reaction times was evaluated. In literature it is reported that basic pH favours the MR, while at acidic pH the reaction is slowed down [12]. The production of 10 KAMs and 5-hydroxymethylfurfural (HMF) was monitored every 20 min in amino acid and reducing sugar solutions at pH 6, 7, and 8 using HRMS. HMF was evaluated because it is a known MRP considered to be a health hazard [13], and its production is considered negative. The samples were compared by analysing the areas of the integrated peaks corresponding to the analytes of interest. As reported in Figure 2, at pH 6, the lowest production of antioxidant compounds but the highest production of HMF was detected. This result agrees with previous literature where HMF production is reported to be induced by acidic pH [14]. The low development of antioxidant compounds at pH 6 increased at pH 7 and 8. In particular, most of the analysed antioxidant compounds show similar kinetics of production in the first part of the reaction (40 min) at pH 7 and 8. This result suggests that their mechanism of formation is favoured by neutral and basic pH. Although, some of them, like DDMP (dihydro-dihydroxymethylpyrone), norfuraneol, and maltol isomer, seem to decrease in concentration after the first part of the reaction, particularly at basic pH. This could indicate that these compounds are MR intermediates and further react and degrade, especially at basic pH, where the reaction is favoured. The development of some other KAMs, like sotolon and maltol, seems to be more induced by neutral pH than basic.

### 2.3. Influence of Amino Acids and Sugars on Antioxidant Production in MR

The influence of amino acids and sugars on the production of KAMs was evaluated keeping the pH constant at 7 and testing all the 20 amino acids and 6 sugars in binary combinations. All the samples obtained were analysed using HPLC-HRMS, and the peaks corresponding to the *m/z* signals of the KAMs and HMF were integrated to obtain areas for comparison. 

**Figure 3:**
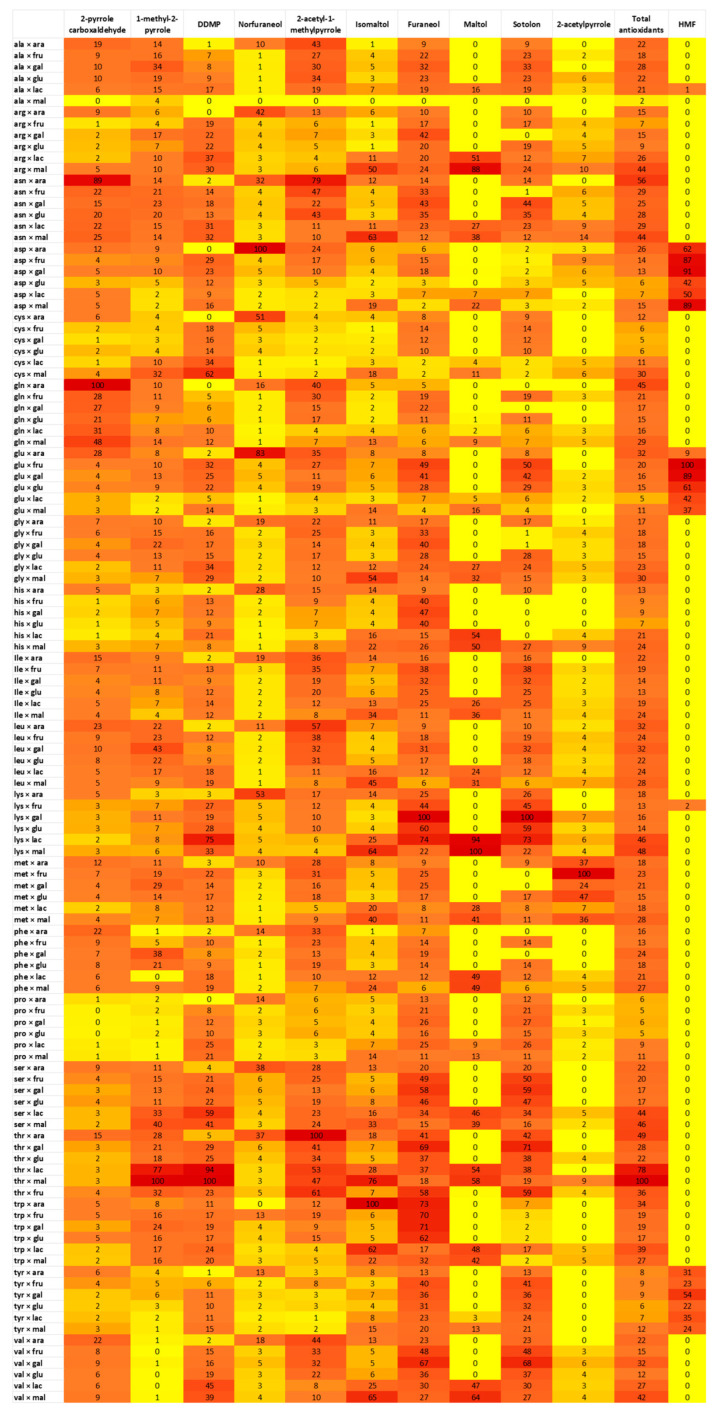
Heatmap representing normalised values of peak areas obtained from HRMS analyses corresponding to *m/z* signals of KAMs for all binary combinations of amino acids and sugars studied. Colours range from red (high value) to yellow (low value). Values are normalised based on the highest for each KAM. Experiments were performed in duplicate; RSD lower than 20%.

Figure 3 displays the normalised values for each antioxidant compound, where the highest recorded area for each compound is set to 100, and the areas for all other samples are normalised relative to this maximum. The values are color-coded based on their numerical value. The experiments were performed in duplicate, and the relative standard deviation (RSD) measured was lower than 20%.

Here are some observations on the analysis of this heatmap. Firstly, it is evident that HMF is mainly produced in the presence of acidic reagents such as glutamic and aspartic acid. This result agrees with previous findings [12]. Secondly, the combination of amino acids and sugars that allowed the highest total production of antioxidants was threonine combined with disaccharides (maltose and lactose). In addition, maltol, as well as the maltol isomer, are mainly produced with disaccharides, while their production is low (maltol isomer) or absent (maltol) with monosaccharides. This result confirms the findings available in the published literature [15].

Moreover, DDMP was produced with all combinations of amino acids and sugars, except for arabinose, the only pentose sugar tested. This suggests that DDMP is an antioxidant MRP produced only with hexose monosaccharides and disaccharides. On the contrary, norfuraneol seems to be mainly produced in the presence of arabinose. Furaneol originates in the presence of tryptophan, while the production of 2-acetyl-1-methylpyrrole is high in MR with threonine and asparagine but low with cysteine, aspartic acid, proline, and tyrosine. 2-pyrrole carboxaldehyde and 1-methyl-2-pyrrole are produced in all combinations, but not with proline (both) and with tyrosine and valine (1-methyl-2-pyrrole). Sotolon shows a peak of production with lysine as the amino acid, even if it is produced with almost all other amino acids. Finally, 2-acetylpyrrole is highly produced in the reaction with methionine but is almost not present with all other amino acids. 

**Figure 4:**
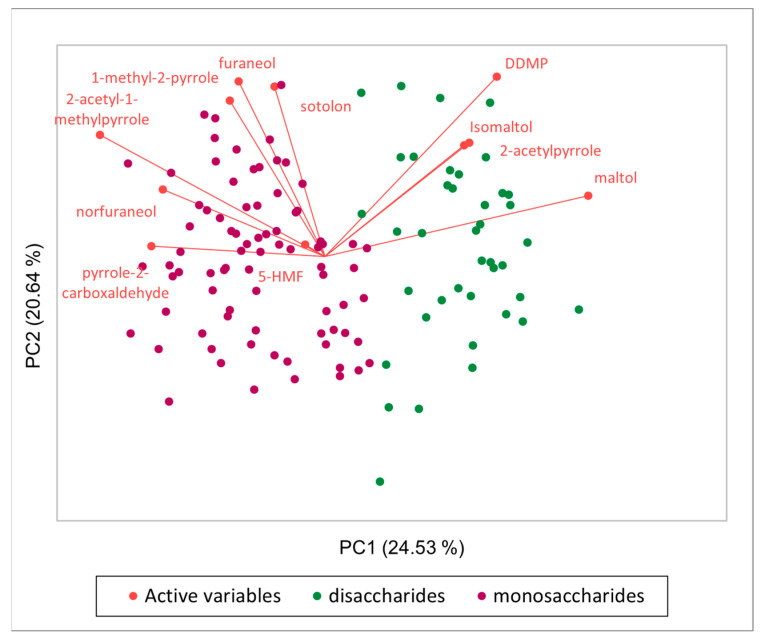
Principal Component Analyses (PCA) of 120 samples with 10 HRMS peak areas corresponding to KAMs and HMF.

In Figure 4**,** the Principal Component Analyses (PCA) of the dataset obtained from the analysis of 120 binary combinations of amino acids and sugars, composed of 11 variables (areas of HRMS peaks corresponding to KAMs and HMF) and 120 observations, is shown. The main differentiation that was obtained regarding the sugar composition, where on the left, samples with monosaccharides (arabinose, fructose, galactose, glucose) are separated from samples containing disaccharides (maltose and lactose), on the right. Moreover, it is possible to understand the variables responsible for this separation, in particular it is evident that maltol, maltol isomer, DDMP, and 2-acetylpyrrole are mainly present in MR samples with disaccharides as reagents, while all the other molecules are characteristic of MR between amino acids andmonosaccharides.

### 2.4. Consumption of Reagents in MR

Sugars and amino acids consumption in a mix sample containing all 20 amino acids and sugars was evaluated to have a more complete understanding of which are the most reactive compounds, and which are less. This information is fundamental to develop further applications of MR as a source of antioxidant compounds.

**Figure 5:**
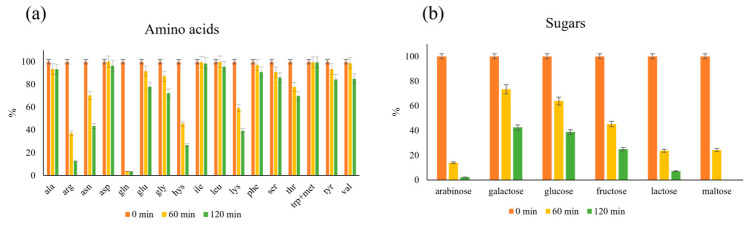
Percentage of consumption of amino acids (**a**) and reducing sugars (**b**) at pH 7 before the MR (0 min) and after 60 and 120 min of MR. Measurements were performed in duplicates, and RSD was lower than 5%.

In Figure 5, the percentage of consumption of amino acids and reducing sugars before 0 min, after 60 min, and 120 min of MR is reported. As evidenced, the most consumed, and therefore reactive amino acids are arginine, asparagine, lysine, and glutamine. This result was expected since it is known that the amino groups of amino acids are the reactive moieties in MR [16], and the most consumed amino acids present amino groups in the side chain. The most consumed sugars, on the other hand, are arabinose, maltose, and lactose. Interesting result is that the disaccharides (lactose and maltose) were consumed more than monosaccharides like glucose and galactose. This aspect can be explained by the thermal degradation of disaccharides at 140 °C, which produces glucose and galactose as products (monosaccharides present in maltose and lactose), thereby increasing their concentration even as they are consumed by MR. 

### 2.5. Untargeted Approach to Create a Library of Potential Antioxidant MRPs

**Figure 6:**
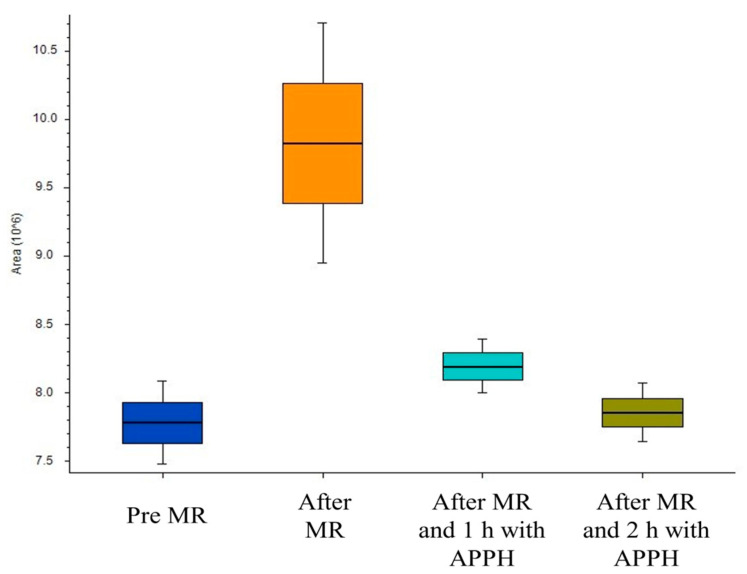
Boxplot used to select the *m/z* signals to create the library of potential antioxidants.

To have a more exhaustive idea of the real potential antioxidant molecules produced through MR, an untargeted approach using HRMS was used. The MR sample obtained from a mix of 20 amino acids and sugars was incubated with a radical initiator (AAPH) to detect, using HRMS, the *m/z* that were produced by MR that also decreased after incubation with the radical initiator. In fact, if a signal decreases in the presence of the radical initiator, it means that the corresponding molecule was oxidised by the radical initiator, suggesting its potential antioxidant properties. Compound Discoverer software was used to analyse the data corresponding to the amino acid and sugar solution before the heating process, after the heating process (and MR), and after 1 and 2 h of incubation with the radical initiator. Figure 6 shows the trend of peak areas during the experiment corresponding to *m/z* of interest. Peaks were selected when their signals were low before MR, increased after MR, and then decreased after incubation with the radical initiator. 

During the creation of the library, the 10 KAMs also resulted showing the same boxplot reported in Figure 6, as expected, and in Table 2 the KAMs are reported, ordered based on the percentage of degradation in the presence of the radical initiator: the higher the percentage of degradation, the quicker the oxidation reaction, and the stronger the antioxidant. Moreover, the best combination of amino acids and sugars to obtain each specific compound is reported, highlighting the possible condition to be followed to produce specific antioxidant MRPs. 

Using this approach, the first library obtained, based only on *m/z* signals and not MS2 fragmentation patterns, included 170 *m/z* signals of potential antioxidants that underwent further selection based on the MS2 fragmentation patterns, obtaining 77 interesting signals. At last, the intensity of signals (areas that were lower than 10^6^ counts * min after MR were excluded) was used to create the final library, which includes 50 *m/z* signals and is available in the supporting information (Table S1).

#### Study of Variable Influence on Antioxidant MRPs Production Using the Library Created

The HRMS spectra obtained from previous experiments (Section 2.2 and Section 2.3) were reprocessed with Compound Discoverer software specifically looking for the signals included in the library created. 

**Figure 7:**
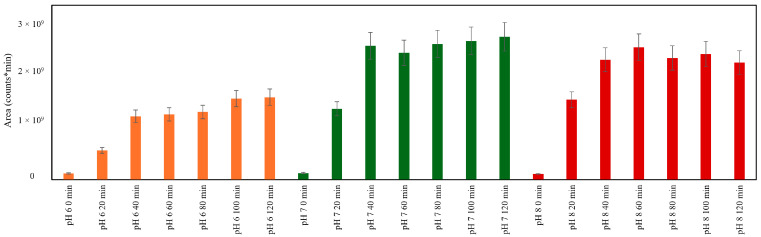
Sum of areas of HRMS peaks corresponding to the 50 *m/z* signals present in the library for each combination of pH and time of MR.

In Figure 7, the total areas of the HRMS peaks corresponding to the 50 *m/z* signals present in the library are reported for each sample at different pHs. It is interesting to note that at pH 7, the highest amount of potential antioxidants is produced, as previously evidenced with the KAMs, but an increase in signals is also observed at pH 6. This result may mean that, even if at pH 6 the KAMs do not develop, probably some other potential antioxidants are produced. 

**Figure 8:**
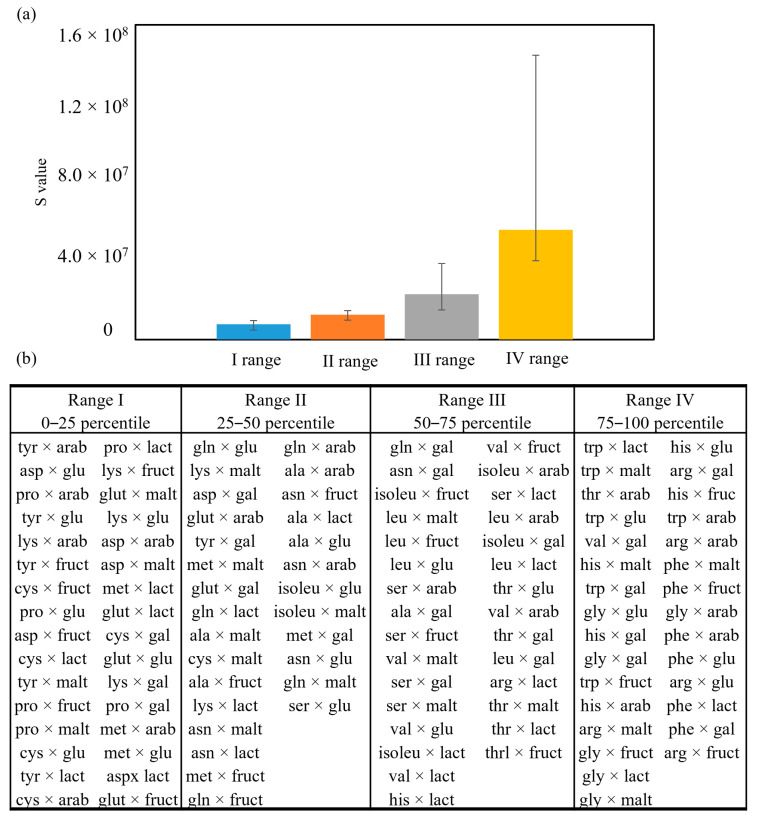
(**a**) S values shown in ranges (range 1: from 0 to 25 percentile; range 2: from 25 to 50 percentile; range 3: from 50 to 75 percentile; range 4: from 75 to 100 percentile) obtained by summing the areas of HRMS peaks corresponding to the 50 *m/z* signals present in the library for each combination of amino acids and sugars, each corrected as described by equation (1). The error bars represent the maximum and minimum values belonging to that range. (**b**) The combinations of amino acids and sugars present in the samples, belonging to ranges I, II, III, and IV.

The spectra corresponding to the binary combinations of amino acids and sugars were also reprocessed, leading to Figure 8a, where S values are shown, divided into ranges (range 1: from 0 to 25 percentile; range 2: from 25 to 50 percentile; range 3: from 50 to 75 percentile; range 4: from 75 to 100 percentile). The table containing all the S values calculated for each sample is available in the Supplementary Material (Table S2). They were obtained by summing the areas of HRMS peaks corresponding to the 50 *m/z* signals present in the library for each combination of amino acids and sugars, each corrected as described by Equation (1). This approach allows the analysis of the combinations of amino acids and sugars, not only considering which pair produces the highest amount of antioxidant molecules but also taking into consideration the tendency of degradation of these PAMs in the presence of a radical initiator. The formula used to process the data is reported below:

(1) $$S=\sum \left(\frac{An}{\%Dn}\right)$$

where *S* is the final value corresponding to each sample plotted in the graph, *An* is the area of each peak recorded with HRMS, and *%Dn* is the percentage of degradation of the peak, calculated as the difference of peak areas * 100 after 1 h of incubation with a radical initiator.

The PAMs present in samples belonging to range I are the ones degrading faster with a radical initiator, compared to the others. PAMs belonging to range IV, instead, are the ones degrading slower, and are, therefore, more stable in presence of a radical initiator. 

As evidenced in Figure 8b, some interesting aspects are revealed. The first one regards the presence of PAMs derived from proline in range I. This means that most of the PAMs produced by proline are very reactive in scavenging radicals and, therefore, quickly degraded. This is interesting because, if compared to the sum of the intensity of the signals produced, not weighted for their % of degradation (available in Figure S2), proline samples are in the average of production, but not the highest producer of PAMs. On the contrary, tryptophane and arginine PAMs belong mostly to range IV, indicating that they are not so reactive with the radical initiator. 

Unfortunately, no other patterns regarding the presence of amino acids or sugars can be drawn based on the production and reactivity of PAMs.

In Table 3, the PAMs included in the library are reported, ordered based on the % of their degradation after 1 h in presence of the radical initiator AAPH. Moreover, the best combination of amino acid and sugar to obtain each compound, based on the data previously presented, is reported. These results give an idea of the optimal conditions to be maintained if the production of one of the specific PAMs is the aim.

## 3. Materials and Methods

### 3.1. Analytical Methods

#### 3.1.1. HPLC-HRMS Analyses

The samples were analysed using a Dionex UltiMate3000 HPLC system (Thermo Fisher Scientific, Waltham, MA, USA) equipped with two binary pumps and an autosampler with a temperature control system. The detector used was a Q-Exactive Orbitrap high-resolution mass spectrometer (HRMS, Thermo Fisher Scientific, Waltham, MA, USA). The heated electrospray source (HESI) was operated in positive and negative ionisation mode with a capillary voltage of 2.50 kV and a capillary temperature of 330 °C. The full MS scan was acquired from 50 to 750 *m/z* with a resolution of 70.000 full width at half maximum (FWHM) at 200 *m/z*, an automatic gain control (AGC) target of 3 × 10^6^ and a maximum injection time of 100 ms. Data-dependent MS analyses (MS2) were conducted to obtain the fragmentation patterns of targeted and untargeted species. In this case, the AGC target was set at 1 × 10^5^, the maximum injection time was 50 ms, and the resolution was 17,500 FWHM with an isolation window of 4.0 *m/z*. The spectrometer was calibrated prior to analyses with Pierce LTQ Velos ESI-positive and negative calibration solution (Thermo Fisher Scientific, Waltham, MA, USA). The data were collected and analysed using Chromeleon 7.3, Compound Discoverer 3.3.3.200, and Mass Frontier 8.3 software (ThermoFisher Scientific, Waltham, MA, USA). 

#### 3.1.2. Amino Acids Quantification

Amino acids were quantified using HPLC coupled with a fluorescence detector (FLD) after derivatisation with ortho-phthalaldehyde (OPA) as described by [17]. Briefly, the measurements were performed using an HPLC 1260 Infinity system (Agilent Technologies, Santa Clara, CA, USA) equipped with a fluorescence detector (Ex = 336 nm, Em = 445 nm). Separation was carried out with sodium acetate 0.05 M (pH 6.9; eluent A) and methanol (eluent B) using a Chromolith Performance RP-18e 100 × 4.6 mm column (Merck, Darmstadt, Germany) with a Guard Cartridge Chromolith RP-18e 10 × 4.6 mm (Merck, Darmstadt, Germany) at 40 °C. The flow rate was set at 2 mL·min^−1^. The analytical gradient for eluent B was as follows: 0% from 0–1 min, 20% from 1–11 min, 40% from 11–16 min, 100% from 16–25 min, 10% from 25–27 min, 0% from 27–30 min. The samples (10 μL), after proper dilution (1:50) with Milli-Q water, adjustment of pH to 7, and filtration with 0.2 nm polytetrafluoroethylene (PTFE) filters, were kept at 10 °C by the autosampler. The derivatisation was automatically carried out by the instrumentation, with the introduction of 10 μL of sample into the loop, addition of 10 μL derivatising solution, mixing for 1 min, and injection. The derivatising mix was 4.5 g·L^−1^ of OPA (Sigma-Aldrich, St. Louis, MO, USA) in sodium tetraborate 0.1 M, adjusted to pH 10.5, 10% methanol, and 2% 2-mercaptoethanol. Agilent OpenLab CDS 3.1 software was used for data acquisition and processing. Quantification was obtained with external amino acids standard calibration curves and internal standard (β-glutamic acid) as a control. 

#### 3.1.3. Sugars Quantification 

The chromatographic separation and quantification of sugars were performed according to the method developed by [18]. The system used was an ICS 5000 ion chromatographer (Dionex, Thermo Fisher Scientific, Waltham, MA, USA) equipped with an eluent generator, an autosampler, a quaternary gradient pump, a column oven, and a pulsed amperometric detector (PAD) consisting of a gold working electrode and a palladium reference electrode. Separation of sugars (both monosaccharides and disaccharides) was performed by injecting 5 µL of sample onto a CarboPac PA200 3 × 250 mm analytical column, preceded by a CarboPac PA200 3 × 50 mm guard column (Dionex, Thermo Fisher Scientific, Waltham, MA, USA). The column stationary phase consisted of a hydrophobic polymeric pellicular resin bonded to quaternary ammonium as an anion-exchange resin functional group. Both columns were operated at a constant temperature of 30 °C. The flow rate was adjusted to 0.4 mL·min^−1^ using an eluent generator that allowed the automatic preparation of potassium hydroxide (KOH) eluent by controlling the electrical current applied for the electrolysis of deionised water. Isocratic KOH elution at 0.1 mM was carried out from 0 to 18 min, followed by gradient elution from 0.1 to 100 mM from 18 to 21.5 min, and held until 27.5 min. The KOH concentration was then reduced to 0.1 mM, allowing the column to equilibrate for 5 min. Deionised water was continuously purged with helium to avoid the formation of carbonates. Sugar detection was performed using PAD with the working pulse potential quaternary curve (Table 4) with respect to a palladium reference electrode.

### 3.2. Sample Preparation and Maillard Reaction

In all the MR experiments, 5 mL of each sample were placed in a 10 mL Pyrex flask sealed with a PTFE hermetic plug and heated in an oven set at 140 °C. This temperature was chosen because it is sufficient to guarantee the active form of the sugars, with an open chain, allowing a faster reaction [19]. The samples were then cooled in an ice bath and kept at −20 °C prior to analyses for a maximum of one week.

#### 3.2.1. Optimisation of Chromatographic Method

The optimisation of the chromatographic method to analyse the antioxidant compounds produced by MR consisted mainly of testing different HPLC columns using as reference the HRMS signals of 10 known antioxidant molecules produced by MR on a mix of amino acids and sugars. The columns and eluents were selected based on the chemical characteristics of the known antioxidant molecules. 

The columns tested were: Acclaim Trinity P1 3 µm 2.1 × 100 mm (Thermo Fisher Scientific, USA); Raptor Biphenyl 2.7 µm 3 × 150 mm (Restek Corporation, Bellefonte, PA, USA); Ionpac NS2 5 µm 4 × 150 mm (Thermo Fisher Scientific, Waltham, MA, USA); Eclipse XDB-C8 µm 4.6 × 150 mm (Agilent Technologies, Santa Clara, CA, USA); and Poroshell 120 HILIC-Z 2.7 µm 2.1 × 100 mm (Agilent Technologies, Santa Clara, CA, USA). 

The samples analysed were obtained by heating for 90 min at 140 °C a water solution containing equimolar concentrations of 20 amino acids (1.25 mM), equimolar concentrations of 6 sugars (4 mM), and phosphate salts to obtain a phosphate buffer solution (PBS) 0.1 M, pH 7. The concentrations of reagents were chosen to achieve a comparable amount of reactive amino groups from amino acids and reactive hydroxyl groups from sugars. Since acid compounds are produced during the MR, the pH level usually decreases [12]; therefore, a buffer solution was used to keep the pH as constant as possible. 

#### 3.2.2. Influence of Initial pH on Antioxidants Production in MR

Once the chromatographic method to detect and separate the KAMs was optimised, the influence of initial pH on antioxidant production in MR was investigated. Three different initial pH were tested: pH 6, pH 7, and pH 8. PBS 0.1 M was used to maintain the pH as constant as possible. Experiments were performed in duplicate. The solutions were prepared in bulk (50 mL), and then six Pyrex flasks with hermetic plugs for each sample were filled with 5 mL. The samples were heated at 140 °C for 120 min, and every 20 min a sample was collected and analysed to evaluate antioxidant and 5-hydroxymethylfurfural (HMF) production. HMF was evaluated because it is a known MRP considered to be a health hazard [13], and its production is considered as negative. 

#### 3.2.3. Influence of Amino Acids and Sugar Composition on Antioxidant Production in MR

The influence of sugar and amino acid composition on KAMs production was evaluated. The amino acids and sugars tested, the most common in food, are reported in Table 5. Since only reducing sugars react in MR [20], non-reducing sugars like sucrose and trehalose were not tested, even though they are common in food samples.

All the amino acids and sugars were combined one-to-one, for a total of 120 different combinations. All the experiment were conducted in PBS 0.1 M at pH 7, with equimolar concentrations of amino acids and sugars (25 mM), except for tyrosine, which was tested at a concentration of 2 mM (always equimolar with sugars) due to its low solubility in water. The samples were kept at 140 °C for 90 min and then cooled and analysed with HPLC coupled with HRMS to detect antioxidant compounds and HMF. 

#### 3.2.4. Consumption of Reagents in MR

Amino acid and sugar consumption (with respect to the non-heated sample) was evaluated in MR samples obtained by heating in an oven at 140 °C a mix solution of all 20 amino acids and 6 sugars at pH 7 for 60 min and 120 min to evaluate which compounds are the most reactive. The mix solution contained equimolar concentrations of 20 amino acids (1.25 mM), equimolar concentrations of 6 sugars (4 mM), and phosphate salts (PBS 0.1 M, pH 7).

#### 3.2.5. Untargeted Approach to Create a Library of Potential Antioxidant MRPs

The MR is a very complex reaction, and MRPs are a wide group of molecules with different bioactivity. Because of these, to obtain a more comprehensive idea of the influence of the variable studied on the production of PAMs, an untargeted approach was used to create a library of *m/z* that represents the main potential molecules produced by MR. To do so, the MR samples were incubated with a radical initiator (AAPH) that activated the oxidation of all potential antioxidants. In a 10 mL volumetric flask, 1 mL of MR sample was added to 100 mM of 2,2′-Azobis(2-methylpropionamidine) dihydrochloride (AAPH) radical initiator in water. The mixture was then separated into four 2 mL vials and incubated at 37 °C for 2 h, taking a sample after 1 h. At 37 °C, the radical initiator starts the oxidation process, and the potential antioxidant compounds are oxidised. This method is based on a previously published study [21] that demonstrated that the peak areas of compounds with potential antioxidant activity in HPLC chromatograms are significantly reduced or disappear after incubation with AAPH, which can release ROO۰ at 37 °C [21]. Once cooled in ice, the samples were analysed using HPLC coupled with HRMS, and all the spectra were processed using Compound discover software 3.3.3.200 (Thermo Fischer Scientific). 

The library of *m/z* obtained was then used to reprocess, using Compound discover software, all the spectra and information obtained from previous experiments (Section 3.2.2 and Section 3.2.3). This approach allowed to obtain a clearer, even if complex, idea of how reaction variables like pH and reagent composition influence antioxidant production in MR. 

## 4. Conclusions

In conclusion, this study elucidated the significant influence of some key variables in MR, such as pH, time, and reagent composition, on antioxidant compound production through the development of a method for the detection and quantification of these compounds. The way for more precise and comprehensive analyses of antioxidant MRPs has been paved, and the next crucial step in this research would be to identify within the large group of new MRPs identified here (PAMs), the molecules with the highest antioxidant activity. This will involve isolating MRPs and characterising their structures and mechanisms of action. Such efforts will not only enhance our understanding of the Maillard reaction’s contribution to food chemistry but also open new avenues for the development of functional foods that valorise these natural antioxidants.

## Figures and Tables

**Table 1:** Antioxidant compounds produced by MR and reported in the literature, with exact mass, theoretical and measured *m/z*, ionisation, and literature reference. * Compounds verified with analytical standards. DDMP: dihydro-dihydroxymethylpyrone.

Compound	Theoretical *m/z*	Measured *m/z*	Δppm	Retention Time	Ionisation	Reference
Maltol *	127.03897	127.03859	0.00038	13.27	[M + H]+	[10]
Maltol isomer	127.03897	127.03905	0.00008	10.98	[M + H]+	[10]
2-acetylpyrrole *	110.06004	110.06005	0.00001	18.79	[M + H]+	[10]
Sotolon *	129.05462	129.04928	0.00534	14.56	[M + H]+	[10]
Norfuraneol *	115.03897	115.03938	0.00041	9.25	[M + H]+	[10]
Furaneol	129.05462	129.06004	0.00542	12.62	[M + H]+	[10]
DDMP	145.04954	145.05029	0.00075	10.50	[M + H]+	[10]
2-pyrrolecarboxaldehyde	96.04439	96.04450	0.00011	4.00	[M + H]+	[11]
1-methyl-2-pyrrole carboxaldehyde	110.06004	110.06099	0.00095	6.00	[M + H]+	[11]
2-acetyl-1-methylpyrrole	124.07569	124.07662	0.00093	9.30	[M + H]+	[11]

**Table 2:** KAMs, ordered based on the percentage of degradation after 1 h of incubation with the radical initiator. *m/z*, retention time, and the best amino acid × sugar combination to obtain each specific compound are also reported.

Compound	% of Degradation after 1 h with Radical Initiator	Theoretical *m/z*	Retention Time (min)	Best AA × Sugar
2-pyrrolecarboxaldehyde	88%	96.0444	4	Glutamine × arabinose
Norfuraneol	70%	115.039	9.25	Aspartic acid × arabinose
Furaneol	69%	129.055	12.62	Lysine × galactose
1-methyl-2-pyrrole carboxaldehyde	60%	110.06	6	Threonine × maltose
DDMP	42%	145.05	10.5	Threonine × maltose
2-acetyl-1-methylpyrrole	38%	124.076	9.3	Threonine × arabinose
Maltol	35%	127.039	13.27	Lysine × maltose
Maltol isomer	31%	127.039	10.98	Tryptophan × arabinose
2-acetylpyrrole	29%	110.06	18.79	Methionine × fructose
Sotolon	13%	129.055	14.56	Lysine × galactose

**Table 3:** PAMs, ordered based on the percentage of degradation after 1 h of incubation with radical initiator. *m/z*, retention time, and the best amino acid × sugar combination to obtain each specific compound are also reported.

Name	% Degradation after 1 h with AAPH	*m/z*	RT [min]	Best AA × Sugar
Unknown 1	96.2	230.1140	8.41	Arginine × fructose
Unknown 2	93.0	199.1077	10.36	Asparagine × fructose
Unknown 3	92.6	154.0497	4.39	Glycine × arabinose
Unknown 4	91.1	124.0757	10.12	Asparagine × glucose
Unknown 5	91.0	164.0818	5.07	Arginine × arabinose
Unknown 6	86.8	170.0811	4.59	Leucine × galactose
Unknown 7	86.0	135.0552	9.21	Histidine × maltose
Unknown 8	83.3	230.1140	6.76	Arginine × fructose
Unknown 9	82.5	212.1028	3.10	Histidine × glucose
Unknown 10	79.7	230.1140	4.13	Arginine × fructose
Unknown 11	79.6	127.0390	6.97	Tryptophan × arabinose
Unknown 12	79.2	151.123	6.65	Leucine × lactose
Unknown 13	77.1	124.0757	8.39	Threonine × fructose
Unknown 14	74.8	140.0706	4.61	Threonine × arabinose
Unknown 15	74.5	119.0350	4.73	Valine × galactose
Unknown 16	73.8	127.0389	3.93	Aspartic acid × lactose
Unknown 17	72.1	154.0498	4.72	Glycine × arabinose
Unknown 18	69.1	212.1034	8.03	Arginine × fructose
Unknown 19	65.0	184.1085	4.72	Arginine × fructose
Unknown 20	64.5	123.0914	3.48	Glycine × fructose
Unknown 21	63.7	135.0553	8.77	Histidine × maltose
Unknown 22	59.7	110.0600	5.15	threonine × maltose
Unknown 23	58.1	166.0861	10.08	Phenylalanine × lactose
Unknown 24	56.2	124.0756	10.56	Asparagine × arabinose
Unknown 25	53.8	127.0389	10.12	Tryptophan × maltose
Unknown 26	53.6	123.0915	3.71	Glycine × fructose
Unknown 27	53.1	110.0600	5.61	Threonine × arabinose
Unknown 28	51.7	230.1141	5.53	Arginine × galactose
Unknown 29	51.6	196.0224	7.01	Glutamine × lactose
Unknown 30	49.9	110.0600	6.38	Leucine × galactose
Unknown 31	47.3	164.0817	6.20	Histidine × fructose
Unknown 32	47.1	143.0349	3.78	Glutamic acid × lactose
Unknown 33	42.0	144.0807	15.71	Tryptophan × galactose
Unknown 34	39.5	124.0757	8.06	Threonine × arabinose
Unknown 35	39.3	124.0756	4.34	Alanine × maltose
Unknown 36	38.2	210.1127	8.29	Threonine × maltose
Unknown 37	37.6	124.0756	15.82	Alanine × fructose
Unknown 38	35.6	169.0972	3.27	Serine × galactose
Unknown 39	34.4	127.039	3.76	Serine × lactose
Unknown 40	31.3	124.0757	7.49	Glutamine × fructose
Unknown 41	30.0	212.1035	6.82	Arginine × fructose
Unknown 42	28.3	217.0971	17.69	Tryptophan × arabinose
Unknown 43	25.5	144.0807	13.62	Tryptophan × galactose
Unknown 44	25.0	144.0807	17.69	Tryptophan × arabinose
Unknown 45	22.0	124.0757	8.63	Asparagine × arabinose
Unknown 46	21.0	143.0350	6.95	Tryptophan × arabinose
Unknown 47	20.1	230.1139	8.12	Arginine × fructose
Unknown 48	14.9	95.06033	3.11	Glycine × galactose
Unknown 49	12.2	151.1229	6.06	Valine × galactose
Unknown 50	10.0	143.035	5.25	Methionine × fructose

**Table 4:** PAD potentials and duration with Ag/AgCl electrode as reference.

Time (s)	Potential (V vs. Ag/AgCl)	Integration
0.00	1.35	Off
0.20	1.35	On
0.40	1.35	Off
0.41	−1.15	Off
0.42	−1.15	Off
0.43	1.45	Off
0.44	1.15	Off
0.50	1.15	Off

**Table 5:** Amino acids and reducing sugars tested, with abbreviations.

Amino Acids			Reducing Sugars
Arginine (arg)	Histidine (his)	Lysine (lys)	Glucose (glu)
Aspartic acid (asp)	Glutamic acid (glu)	Serine (ser)	Galactose (gal)
Threonine (thr)	Asparagine (asn)	Glutamine (gln)	Fructose (fru)
Cysteine (cys)	Glycine (gly)	Proline (pro)	Arabinose (ara)
Alanine (ala)	Valine (val)	Isoleucine (Ile)	Maltose (mal)
Leucine (leu)	Methionine (met)	Phenylalanine (phe)	Lactose (lac)
Tyrosine (tyr)	Tryptophan (trp)		

## Data Availability

The authors confirm that the original contributions presented in the study are included in the article/Supplementary Material ; further inquiries can be directed to the corresponding author/s.

## Supplementary Materials

The following supporting information can be downloaded at: https://www.mdpi.com/article/10.3390/molecules29204820/s1. Figure S1: Extracted ion chromatograms (EIC) referring to *m/z* of known antioxidant MRPs are reported for all the tested and discarded columns; Table S1: *m/z* PAMs library with retention times, reference ion and main MS2 fragments when available; Table S2: S values obtained as described in the manuscript (eq. 1) corresponding to each sample and divided in ranges based on the percentile; Figure S2: Sum of areas of HRMS peaks corresponding to the 50 *m/z* signals present in the library for each combination of amino acids and sugars. In yellow, the samples with amino acids that contain a nitrogen atom in the side chain; in blue, all the others.

## References

[1] Nooshkam M., Varidi M., Bashash M. (2019). The Maillard reaction products as food-born antioxidant and antibrowning agents in model and real food systems. Food Chem..

[2] Salter L.J., Mottram D.S., Whitfield F.B. (1989). Volatile compounds produced in maiilard reactions involving glycine, ribose and phospholipid. J. Sci. Food Agric..

[3] Sun A., Wu W., Soladoye O.P., Aluko R.E., Bak K.H., Fu Y., Zhang Y.H. (2022). Maillard reaction of food-derived peptides as a potential route to generate meat flavor compounds: A review. Food Res. Int..

[4] Michalska A., Amigo-Benavent M., Zielinski H., del Castillo M.D. (2008). Effect of bread making on formation of Maillard reaction products contributing to the overall antioxidant activity of rye bread. J. Cereal Sci..

[5] Nooshkam M., Varidi M., Verma D.K. (2020). Functional and biological properties of Maillard conjugates and their potential application in medical and food: A review. Food Res. Int..

[6] Wang H.Y., Qian H., Yao W.R. (2011). Melanoidins produced by the Maillard reaction: Structure and biological activity. Food Chem..

[7] Novais C., Molina A.K., Abreu R.M.V., Santo-Buelga C., Ferreira I.C.F.R., Pereira C., Barros L. (2022). Natural Food Colorants and Preservatives: A Review, a Demand, and a Challenge. J. Agric. Food Chem..

[8] Fu Y., Zhang Y., Soladoye O.P., Aluko R.E. (2020). Maillard reaction products derived from food protein-derived peptides: Insights into flavor and bioactivity. Crit. Rev. Food Sci. Nutr..

[9] Liu X., Xia B., Hu L.T., Ni Z.J., Thakur K., Wei Z.J. (2020). Maillard conjugates and their potential in food and nutritional industries: A review. Food Front..

[10] Kanzler C., Haase P.T., Schestkowa H., Kroh L.W. (2016). Antioxidant Properties of Heterocyclic Intermediates of the Maillard Reaction and Structurally Related Compounds. J. Agric. Food Chem..

[11] Yanagimoto K., Lee K.G., Ochi H., Shibamoto T. (2002). Antioxidative activity of heterocyclic compounds formed in Maillard reaction products. Int. Congr. Ser..

[12] Martins S.I.F.S., Van Boekel M.A.J.S. (2005). Kinetics of the glucose/glycine Maillard reaction pathways: Influences of pH and reactant initial concentrations. Food Chem..

[13] Zhang L., Kong X., Yang X., Zhang Y.Y., Sun B.G., Chen H.T., Sun Y. (2019). Kinetics of 5-hydroxymethylfurfural formation in the sugar—Amino acid model of Maillard reaction. J. Sci. Food Agric..

[14] Delgado-andrade C., Seiquer I., Haro A., Castellano R., Navarro M.P. (2010). Development of the Maillard reaction in foods cooked by different techniques. Intake of Maillard-derived compounds. Food Chem..

[15] Yaylayan V.A., Mandeville S. (1994). Stereochemical Control of Maltol Formation in Maillard Reaction. J. Agric. Food Chem..

[16] Amaya-Farfan J., Rodriguez-Amaya D.B. (2021). The Mailard Reactions. Chemical Changes During Processing and Storage of Foods.

[17] Gallo A., Guzzon R., Ongaro M., Paolini M., Nardin T., Malacarne M., Roman T., Larcher R. (2023). Biological acidification of ‘Vino Santo di Gambellara’ by mixed fermentation of L. thermotolerans and S. cerevisiae. Role of nitrogen in the evolution of fermentation and aroma profile. Oeno. One.

[18] Di Lella S., Tognetti R., La Porta N., Lombardi F., Nardin T., Larcher R. (2019). Characterization of Silver fir Wood Decay Classes Using Sugar Metabolites Detected with Ion Chromatography. J. Wood Chem. Technol..

[19] Van Boekel M.A.J.S. (2001). Kinetic aspects of the Maillard reaction: A critical review. Food/Nahrung.

[20] Hodge J.E. (1953). Browning Reaction Theories Integrated in Review Chemistry of Browning Reactions in Model Systems. Agric. Food Chem..

[21] Zhuang G.D., Gu W.T., Xu S.H., Cao D.M., Deng S.M., Chen Y.S., Wang S.M., Tang D. (2023). Rapid screening of antioxidant from natural products by AAPH-Incubating HPLC-DAD-HR MS/MS method: A case study of Gardenia jasminoides fruit. Food Chem..

