# Supplementary material for: Screening of Antioxidant Maillard Reaction Products Using HPLC-HRMS and Study of Reaction Conditions for Their Production as Food Preservatives
Source: Molecules. 2024 Oct 11;29(20):4820. doi: 10.3390/molecules29204820 (PMC11510528; doi:10.3390/molecules29204820)

Figure S1: extracted ion chromatograms (EIC) referring to *m/z* of known antioxidant MRPs, are reported for all the tested and discarded columns.

Column: Acclaim Trinity P1 3 µm 2.1x100 mm (Thermo Fischer Scientific, USA)

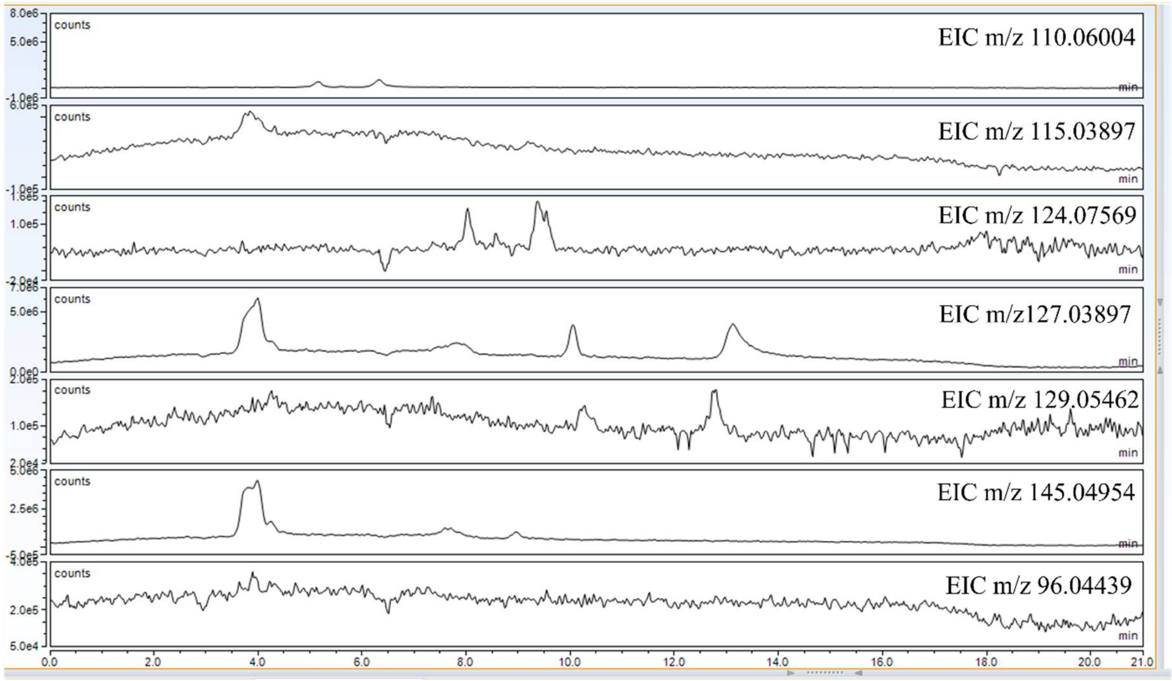

Column: Raptor Biphenyl 2.7 µm 3x150 mm (Restek Corporation, USA)

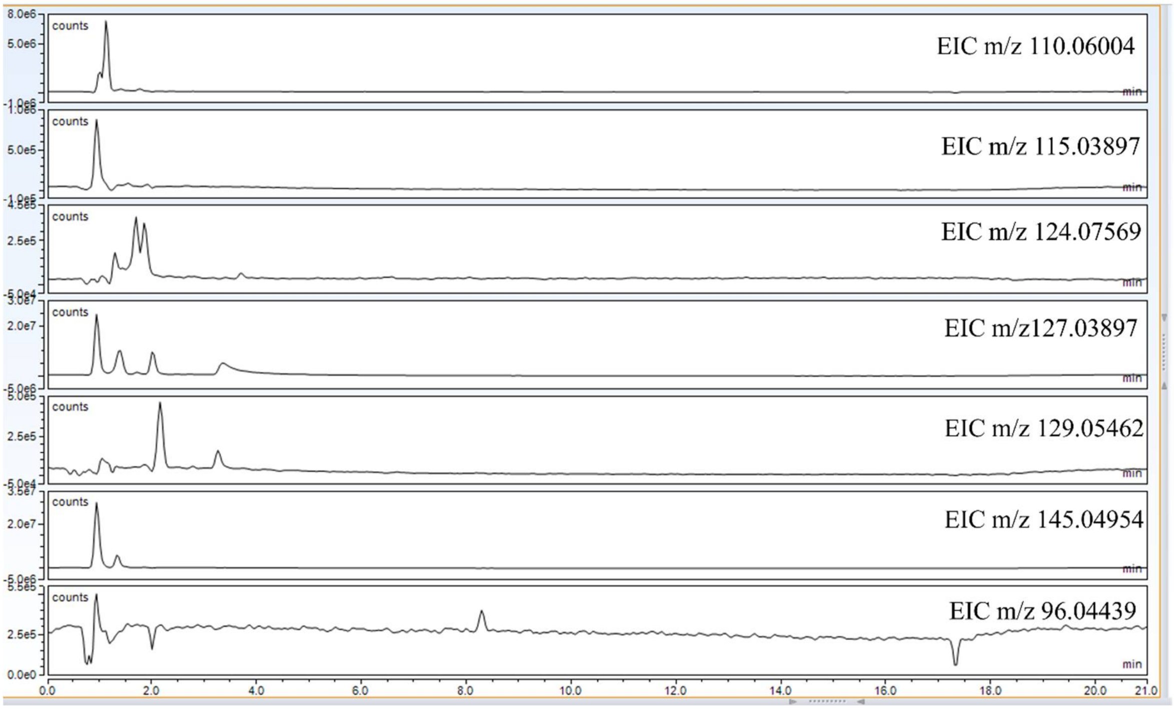

Column: Eclipse XDB-C8 µm 4.6x150 mm (Agilent Technologies, USA)

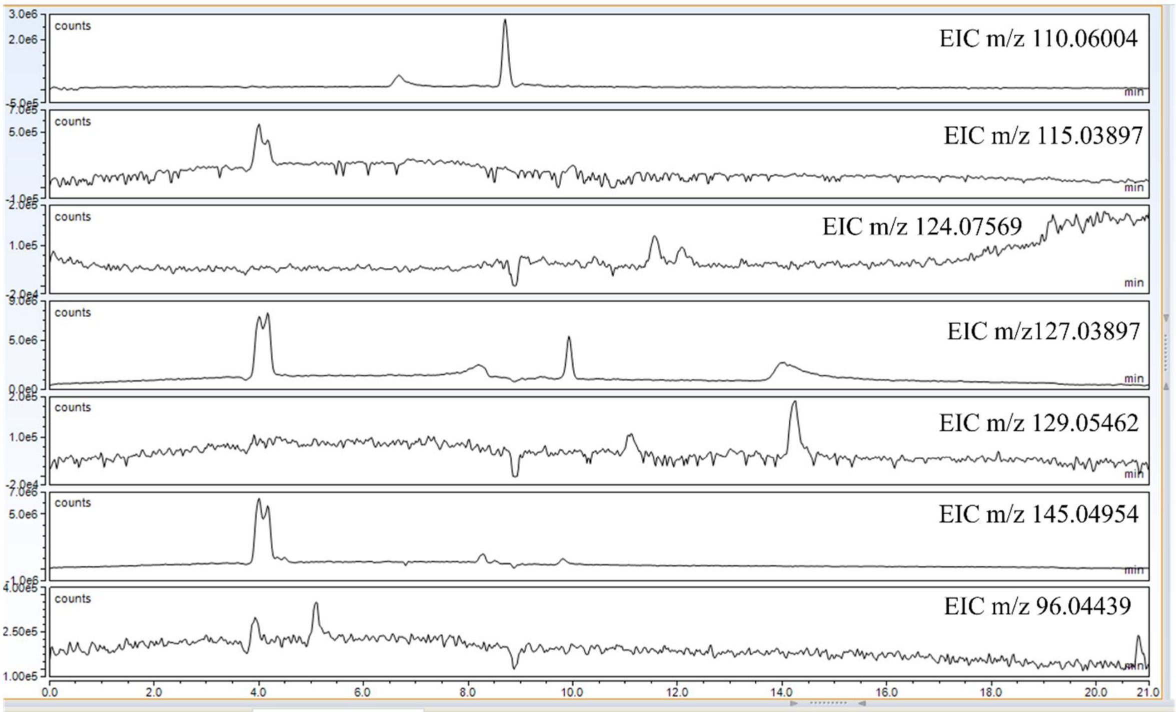

Column: Poroshel 120 HILIC-Z 2.7 µm 2.1x100 mm (Agilent Technologies, USA)

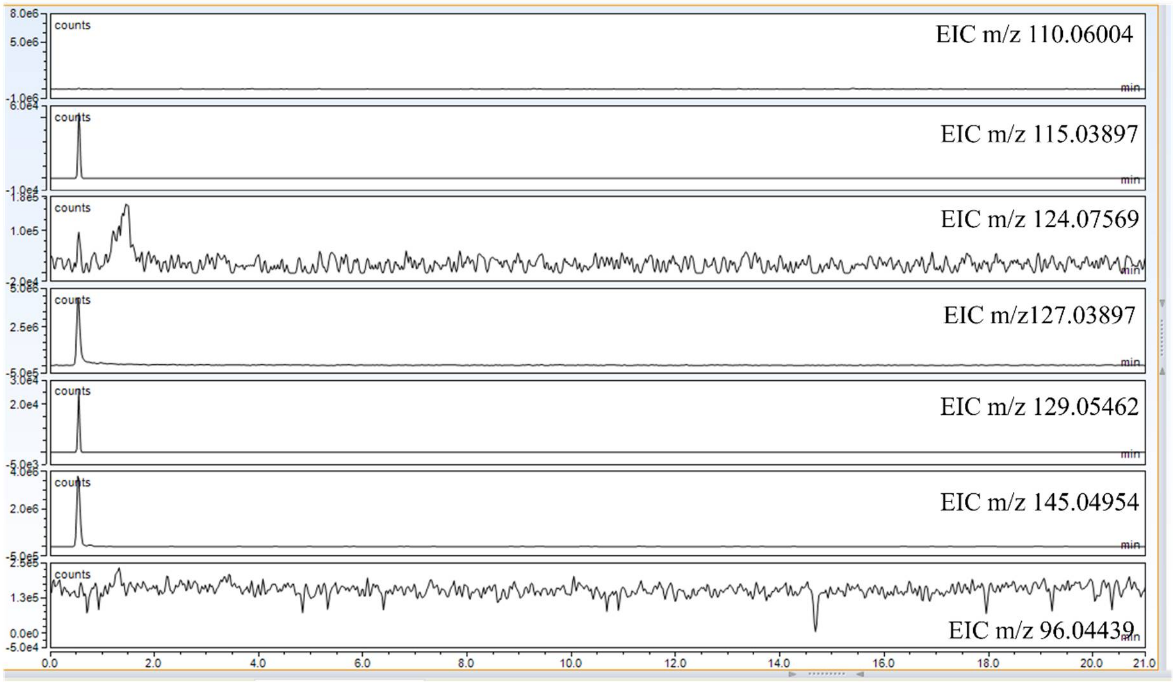

Table S1: *m/z* PAMs library with retention times, reference ion and main ms2 fragments when available

Name	m/z	RT [min]	Reference Ion	Fragments ms2
Unknown 1	230.11395	6.764	[M+H]+1	70.0653, 149.0711, 184.1082

Unknown 2	199.10773	10.363	[M+H]+1	109.0761, 152.0820, 177.9567
Unknown 3	154.04974	4.392	[M+H]+1	n.a.
Unknown 4	124.07567	10.12	[M+H]+1	84.9599, 95.0493, 123.0806
Unknown 5	164.08179	5.078	[M+H]+1	116.9519, 118.9675, 146.0715
Unknown 6	170.08114	4.59	[M+H]+1	109.0649, 134.0602, 152.0708
Unknown 7	135.05519	9.218	[M+H]+1	74.9377, 115.9642, 117.9596
Unknown 8	230.11398	8.414	[M+H]+1	115.0867, 159.0766, 184.1082
Unknown 9	212.10277	3.104	[M+H]+1	n.a.
Unknown 10	230.11401	4.137	[M+H]+1	115.0504, 167.0818, 212.1035
Unknown 11	127.039	10.97	[M-H]-1	92.0202, 94.9172, 136.8631
Unknown 12	151.12297	6.654	[M+H]+1	n.a.
Unknown 13	124.0757	9.39	[M-H]-1	55.9348, 84.9598, 109.0284
Unknown 14	140.07061	4.615	[M+H]+1	98.9843, 116.9723, 122.0601
Unknown 15	119.03497	4.738	[M-H]-1	59.0138, 73.0294, 101.0243
Unknown 16	127.0389	3.93	[M+H]+1	81.0337, 108.0445, 109.0286
Unknown 17	154.0498	4.725	[M+H]+1	n.a.
Unknown 18	212.10344	8.03	[M+H]+1	166.0977, 184.1083, 193.9303
Unknown 19	184.10849	4.724	[M+H]+1	70.0653, 138.1027, 166.0976
Unknown 20	123.09144	3.485	[M+H]+1	62.9293, 95.0493, 121.0398
Unknown 21	135.05527	8.774	[M+H]+1	74.9377, 107.0605, 115.9642
Unknown 22	110.06003	5.15	[M+H]+1	67.0544, 72.9373, 90.9478
Unknown 23	166.08611	10.088	[M+H]+1	103.0544, 120.0809, 131.0493
Unknown 24	124.07566	10.56	[M+H]+1	84.9599, 95.0493, 123.0806

Unknown 21	127.0389	10.12	[M+H]+1	n.a.
Unknown 26	123.09151	3.71	[M+H]+1	62.9293, 95.0493, 121.0398
Unknown 27	110.06003	5.61	[M+H]+1	67.0544, 72.9373, 90.9478
Unknown 28	230.11409	5.538	[M+H]+1	115.0504, 187.9166, 212.1035
Unknown 29	196.0224	7.017	[M-H]-1	n.a.
Unknown 30	110.06004	5.15	[M+H]+1	n.a.
Unknown 31	164.08169	6.205	[M+H]+1	116.9519, 118.9675, 146.0715
Unknown 32	143.03493	3.781	[M-H]-1	71.0138, 87.0086, 100.9534
Unknown 33	144.08066	13.628	[M+H]+1	62.9293, 84.9598, 120.0237
Unknown 34	124.0757	8.06	[M-H]-1	51.0188, 81.0344, 97.0294
Unknown 35	124.07566	4.34	[M+H]+1	53.0391, 84.9598, 95.0492
Unknown 36	210.11266	8.293	[M+H]+1	112.0516, 166.0983, 182.9539
Unknown 37	124.07567	15.82	[M+H]+1	84.9599, 95.0493, 97.0078
Unknown 38	169.09722	3.279	[M+H]+1	n.a.
Unknown 39	127.039	3.76	[M-H]-1	n.a.
Unknown 40	124.0757	7.49	[M+H]+1	81.0337, 108.0445, 109.0286
Unknown 41	212.10347	6.82	[M+H]+1	170.9141, 184.1082, 193.9303
Unknown 42	217.09706	17.692	[M+H]+1	74.0238, 144.0810, 171.0919
Unknown 43	144.08068	15.716	[M+H]+1	62.9293, 84.9598, 121.9663
Unknown 44	144.08068	17.692	[M+H]+1	62.9293, 84.9598, 121.9663
Unknown 45	124.0757	8.63	[M+H]+1	81.0337, 97.0285, 109.0286
Unknown 46	143.03499	5.253	[M-H]-1	71.0137, 100.9533, 113.0242
Unknown 47	230.11393	8.123	[M+H]+1	115.0867, 159.0766, 184.1082

Unknown 48	95.06033	3.117	[M+H]+1	68.0497, 80.0370
Unknown 49	151.12289	6.062	[M+H]+1	71.0857, 81.0449, 113.9639
Unknown 50	143.035	6.956	[M-H]-1	85.9298, 100.9533, 115.9204

Table S2: S values obtained as described in the manuscript corresponding to each sample and divided in ranges based on the percentile.

**0-25 percentile**		**25-50 percentile**		**50-75 percentile**		**75-100 percentile**	
**Sample**		**S value**		**Sample**		**S value**	
**tyr** x arab	**4794408**	**gln** x glu	**9747622**	**gln** x gal	**15079915**	**trp** x lact	**39980841**
asp x glu	4925437	lys x malt	9918086	asn x gal	15424305	trp x malt	40406820
pro x arab	5726284	asp x gal	9946020	isoleu x fruct	16386509	thr x arab	42584139
tyr x glu	5773161	glut x arab	9983438	leu x malt	16479530	trp x glu	43666721
lys x arab	6476942	tyr x gal	10000634	leu x fruct	17786221	val x gal	43785091
tyr x fruct	6508976	met x malt	10011592	leu x glu	17836045	his x malt	44765889
cys x fruct	6788549	glut x gal	10055352	ser x arab	18531156	trp x gal	46452201
pro x glu	7171232	gln x lact	10731095	ala x gal	18897126	gly x glu	47067364
asp x fruct	7312297	ala x malt	10902510	ser x fruct	18990518	his x gal	48033472
cys x lact	7380824	cys x malt	11140066	val x malt	20480883	gly x gal	48982544
tyr x malt	7386135	ala x fruct	11483321	ser x gal	20754619	trp x fruct	49931484
pro x fruct	7429607	lys x lact	11631685	ser x malt	21171675	his x arab	51027926

pro x malt	7473839	asn x malt	12347087	val x glu	21793197	arg x malt	54724534
cys x glu	7496645	asn x lact	12526165	isoleu x lact	22580227	gly x fruct	54840124
tyr x lact	7604976	met x fruct	12582961	val x lact	22841453	gly x lact	55243520
cys x arab	7639243	gln x fruct	12766240	his x lact	23177411	gly x malt	56167509
pro x lact	7720577	gln x arab	13044629	val x fruct	23226973	his x glu	58170816
lys x fruct	7994222	ala x arab	13745606	isoleu x arab	24366748	arg x gal	63619636
glut x malt	8077658	asn x fruct	13812616	ser x lact	26021245	his x fruc	64323567
lys x glu	8304313	ala x lact	13862719	leu x arab	26697798	trp x arab	64760558
asp x arab	8531124	ala x glu	13902176	isoleu x gal	26956998	arg x arab	92308287
asp x malt	8664643	asn x arab	14061766	leu x lact	27661593	phe x malt	1.03E+08
met x lact	8830069	isoleu x glu	14104338	thr x glu	28002896	phe x fruct	1.04E+08
glut x lact	8837138	isoleu x malt	14135791	val x arab	31980370	gly x arab	1.04E+08
cys x gal	8865400	met x gal	14196707	thr x gal	33402492	phe x arab	1.05E+08
glut x glu	9003189	asn x glu	14492620	leu x gal	35453153	phe x glu	1.06E+08
lys x gal	9106060	gln x malt	14560732	arg x lact	35682008	arg x glu	1.08E+08

pro x gal	9132118	ser x glu	14714193	thr x malt	35755829	phe x lact	1.08E+08
met x arab	9211482			thr x lact	36526674	phe x gal	1.11E+08
met x glu	9517490			thrl x fruct	38518878	arg x fruct	1.45E+08
aspx lact	9529438						
glut x fruct	9592808						

Figure S2: sum of areas of HRMS peaks corresponding to the 50 m/z signals present in the library for each combination of amino acids and sugars. In yellow the samples with amino acids that present a nitrogen atom in the side chain, in blue all the others.

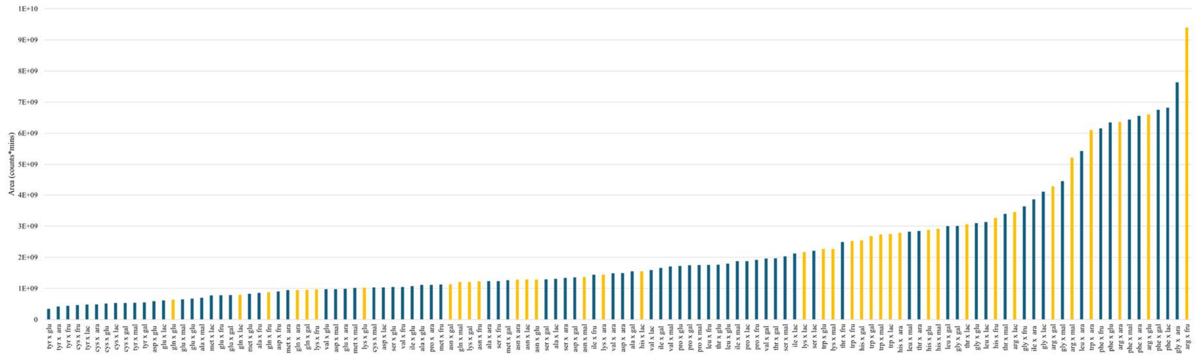

Supplement: Supplementary file 1 [file molecules-29-04820-s001.zip › molecules-3218168-supplementary.pdf]
